# Cefepime continuous infusion as a part of antibiotic stewardship against MDR: NICU of Cipto Mangunkusumo Hospital (CMH) experience

**DOI:** 10.1017/ash.2025.93

**Published:** 2025-09-03

**Authors:** Muhammad Azharry Rully, Putri Maharani Tristanita Marsubrin, Rosalina Dewi Roeslani, Ahmad Kautsar, Nina Dwi Putri, Jessica Sylvania Oswari

**Keywords:** cefepime continuous, efficacy, sepsis, antibiotic stewardship

## Abstract

**Background:** As the incidences of preterm births and surgical cases increases, so do cases of neonatal sepsis in CMH. Furthermore, the common etiology of neonatal sepsis are multidrug-resistant bacteria which increase the risk of mortality. Cefepime is a fourth-generation cephalosporin which is increasingly being utilized in NICUs. Theoretically, continuous infusion of beta lactam antibiotics could maximize the time- dependent bactericidal activity and improve the probability of target attainment. This study aims to determine the effectiveness and safety of continuous cefepime administration in managing sepsis. **Methods:** This is retrospective cohort study on infants who suspected late onset sepsis from 2021 to 2023. The independent variables are continuous infusion and intermittent infusion, with outcomes including mortality rate, reduction in septic markers, use of antibiotic combinations, duration of antibiotic use, and renal function test. **Result:** There were 106 subjects receiving cefepime (56 continuous and 50 intermittent infusions; p>0.05). No significant differences in demographic data such as gestational age, prematurity condition, birth weight, and surgical conditions were found between the two methods. Out of 66 subjects with proven sepsis, 28% were classified as MDR, 12% as XDR, and 16% as PDR. No difference in sepsis-related mortality outcomes was observed between the two methods (64.3% vs. 70%; p=0.532). Continuous administration reduced C-reactive protein (80.52 vs. 51.69 mg/L; p=0.000) and procalcitonin (11.9 vs. 6.72 ng/mL; p=0.008) more effectively than intermittent. In surgical cases, continuous administration reduced the risk of multidrug therapy (RR 0.5 CI 95% 0.243-0.902; p=0.045). There was no difference renal function impairment between two methods. **Conclusion:** Cefepime continuous infusion can significantly reduce infection markers compared to intermittent administration. In surgical cases, continuous cefepime administration reduces the risk of multidrug therapy. The use of continuous cefepime can be considered as part of antibiotic stewardship in the NICU.

